# Identification of Dopamine D2 Receptor as a Direct Target of Salidroside and Tyrosol by Integrated Transcriptomic and Biophysical Approaches

**DOI:** 10.3390/ph19040540

**Published:** 2026-03-27

**Authors:** Jizhou Zhang, Kan Lin, Chang Jiang, Jiabing Zheng, Huihui Huang, Jing Han

**Affiliations:** 1 Institute of Materia Medica, Fujian Academy of Chinese Medical Science, Fuzhou 350003, China; zhangjizhou@fjtcm.edu.cn (J.Z.); fjjc@fjtcm.edu.cn (C.J.); 2 Key Laboratory of Fujian Province Universities on Ion Channel and Signal Transduction in Cardiovascular Diseases, School of Basic Medical Sciences, Fujian Medical University, Fuzhou 350108, China; 3 Fujian Medical University Union Hospital, Fuzhou 350001, China; linkan@fjmu.edu.cn (K.L.); zhengjiabing@fjmu.edu.cn (J.Z.); 4 Fujian Key Laboratory of Drug Target Discovery and Structural and Functional Research, Fujian Medical University, Fuzhou 350108, China; 5 Department of Pharmacology, School of Pharmacy, Fujian Medical University, Fuzhou 350108, China

**Keywords:** salidroside, tyrosol, Connectivity Map database, dopamine D2 receptor, ERK

## Abstract

**Background/Objectives:** Salidroside, a bioactive phenylethanol glycoside primarily derived from Rhodiola rosea, and its major in vivo metabolite tyrosol exhibit diverse pharmacological activities. However, their direct molecular targets remain poorly defined. **Methods:** In the present study, an integrated strategy combining transcriptomic profiling, Connectivity Map (CMap) analysis, and multi-level experimental validation was employed. Transcriptomic signatures derived from A549 cells treated with salidroside or tyrosol were queried against the CMap database. Molecular docking, surface plasmon resonance (SPR), and cellular thermal shift assays (CETSA) were performed to predict and validate binding interactions. Functional validation was performed in SH-SY5Y cells. The phosphorylation level of extracellular signal-regulated kinase (ERK), a downstream signaling event of dopamine D2 receptor (DRD2), was detected after salidroside and tyrosol treatment. DRD2 antagonist sulpiride pre-intervention and small interfering RNA (siRNA)-mediated DRD2 knockdown were conducted to verify the receptor dependence of the compounds’ effects. **Results:** CMap analysis revealed that the transcriptomic signatures of salidroside and tyrosol showed significant similarity to known DRD2 modulators. Molecular docking predicted potential binding interactions between the two compounds and DRD2, which was confirmed by SPR and CETSA to be direct physical binding. Functional studies showed that both compounds rapidly induced DRD2 downstream ERK phosphorylation in SH-SY5Y cells; this effect was abrogated by sulpiride or DRD2 knockdown, indicating DRD2-dependent signaling activation. **Conclusions:** These findings identify DRD2 as a direct molecular target of salidroside and tyrosol and provide mechanistic insight into their dopaminergic regulatory effects. This study highlights the utility of CMap-guided target discovery combined with rigorous experimental validation for elucidating the molecular mechanisms of natural products.

## 1. Introduction

Salidroside is the principal bioactive constituent of the traditional Chinese medicinal herb *Rhodiola rosea* L. and is also present in other medicinal plants such as *Ligustrum lucidum* Ait. As a phenylethanol glycoside, salidroside is rapidly and extensively metabolized in vivo to tyrosol, which represents its major circulating and tissue-distributed form following administration. Tyrosol itself is also a naturally occurring phenolic compound abundant in olive oil and red wine. Pharmacokinetic studies have demonstrated that, after intravenous administration, salidroside is immediately and extensively metabolized to tyrosol, which has been identified as the main form present in all rat tissues, rather than salidroside [1]. Both salidroside and tyrosol belong to the phenylethanol derivative family and exhibit a broad spectrum of biological activities in vivo.

Extensive pharmacological studies have shown that salidroside exerts antioxidant, anti-inflammatory, anticancer, cardioprotective, neuroprotective, antidepressant, anti-aging, antidiabetic, lipid-lowering, and immunomodulatory effects [2,3]. Similarly, tyrosol has been reported to possess antioxidant, anti-inflammatory, anticancer, cardioprotective, neuroprotective, and antidepressant activities [4,5]. At the molecular level, these diverse bioactivities have been associated with the modulation of multiple signaling pathways and regulatory molecules, including nuclear factor kappa-B(NF-κB), tumor necrosis factor-α (TNF-α), adenosine 5′-monophosphate-activated protein kinase (AMPK), phosphatidylinositol 3-kinase/protein kinase B (PI3K/Akt), janus kinase/signal transducer and activator of transcription (JAK/STAT), and mitogen-activated protein kinase kinase/extracellular signal-regulated kinase (MEK/ERK) pathways for salidroside [2,6,7], as well as cyclooxygenase-2 (COX-2), 3-hydroxy-3-methylglutaryl coenzyme A reductase (HMGCoAR), p38 mitogen-activated protein kinase (p38 MAPK), ERK, and α-amino-3-hydroxy-5-methyl-4-isoxazolepropionic acid (AMPA)-related signaling for tyrosol [8,9,10]. Despite these advances, most existing studies have focused on downstream signaling events or pathological models, and the direct molecular targets responsible for the primary actions of salidroside and tyrosol remain largely undefined.

Notably, our previous studies demonstrated that salidroside exerted pronounced neuroprotective effects in a rat model of cerebral ischemia–reperfusion (I/R) injury [11,12]. Using in vivo cerebral microdialysis, we further observed that salidroside administration rapidly increased dopamine levels and its metabolites in the striatum of I/R model rats [13], suggesting a regulatory effect on the dopaminergic nervous system following ischemic insult. These findings raised the possibility that salidroside and its metabolite tyrosol may directly interact with key components of dopaminergic signaling; however, the molecular basis for this regulation has not yet been elucidated.

The Connectivity Map (CMap), first reported in Nature in 2007 [14], is a transcriptome-based pharmacogenomic platform that links gene expression signatures induced by small molecules to those generated by reference compounds with known mechanisms of action. By comparing similarities between transcriptional perturbation profiles, CMap enables the identification of compounds with shared targets or biological activities and has been increasingly applied to drug target identification, drug repurposing, and mechanism-of-action studies [15]. This approach is particularly well suited for natural products and traditional Chinese medicine-derived compounds, whose pharmacological effects are often multifaceted and whose direct molecular targets are difficult to predict based solely on chemical structure.

**Figure 1:**
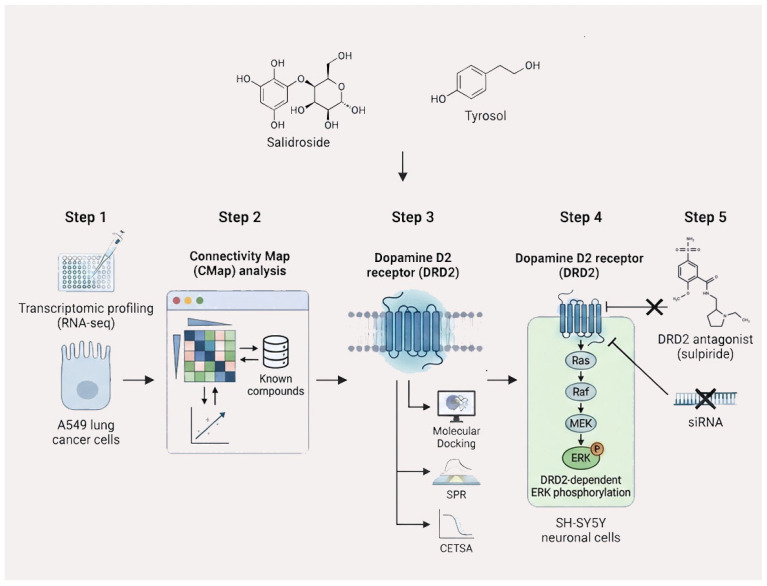
Methodological flowchart for investigating the mechanism of salidroside and tyrosol action mediated by DRD2.

In the present study, we employed RNA sequencing to obtain the transcriptome signatures from A549 cells treated with salidroside and tyrosol. Then the signatures were used to query the CMap database. Based on CMap-guided target prioritization, we further applied a series of complementary validation approaches, including molecular docking, surface plasmon resonance (SPR), cellular thermal shift assay (CETSA) [16], and functional signaling analyses, to determine whether salidroside and tyrosol directly engage the predicted target. This integrated strategy was designed to systematically identify and validate the direct molecular targets of salidroside and its major metabolite tyrosol. The process flow of the study, detailing each step from chemical analysis to experimental verification, is clearly depicted in Figure 1.

## 2. Results

### 2.1. RNA Sequencing

**Figure 2:**
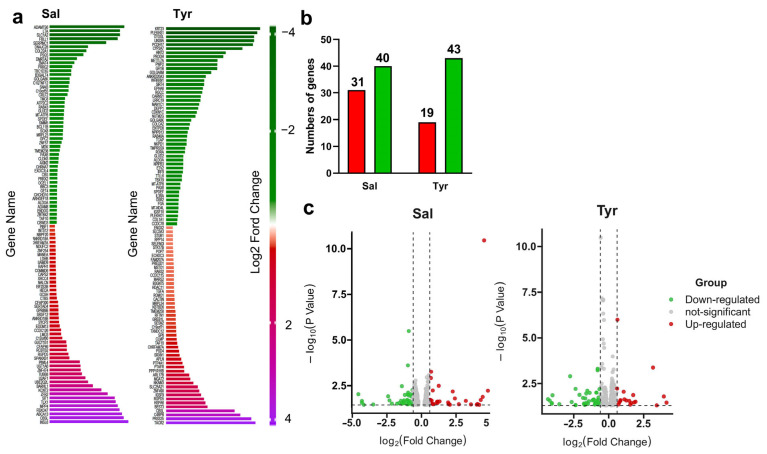
Transcriptomic profiling of A549 cells treated with salidroside or tyrosol. (**a**) Heatmaps showing the top 50 upregulated and top 50 downregulated protein-coding genes in A549 cells following treatment with salidroside or tyrosol (60 μM, 12 h) compared with control cells. Gene expression levels are displayed as normalized values, with red indicating higher expression and green indicating lower expression. Only the top 100 differentially expressed genes are shown; the complete list of DEGs is provided in Supplementary File S1. (**b**) Numbers of differentially expressed genes identified in salidroside- and tyrosol-treated cells relative to control cells, using an adjusted *p* value < 0.05 and a fold change threshold of ≥1.5 (|log_2_FC| ≥ 0.6). (**c**) Volcano plots illustrating the global distribution of differentially expressed genes in response to salidroside or tyrosol treatment. Red dots represent significantly upregulated genes, green dots represent significantly downregulated genes, and gray dots indicate genes without significant changes.

To characterize the transcriptional responses induced by salidroside and tyrosol, A549 cells were treated with salidroside or tyrosol (60 μM) for 12 h, followed by RNA sequencing analysis. Differentially expressed genes (DEGs) were identified by comparing treated cells with control cells. The top 50 upregulated and downregulated protein-coding genes in response to salidroside or tyrosol treatment are shown in the heatmaps in Figure 2a.

Using an adjusted *p* value < 0.05 and a fold change threshold of 1.5 (|log2FC| ≥ 0.6) as the screening criteria, the total numbers of DEGs induced by salidroside and tyrosol were determined (Figure 2b). Volcano plots illustrating the global distribution of upregulated and downregulated genes are shown in Figure 2c. A complete list of DEGs is provided in Supplementary File S1. These results demonstrate that salidroside and tyrosol elicit robust and distinct transcriptional responses, indicating their broad regulatory effects on cellular gene expression.

### 2.2. GO Enrichment Analysis of the DEGs

**Figure 3:**
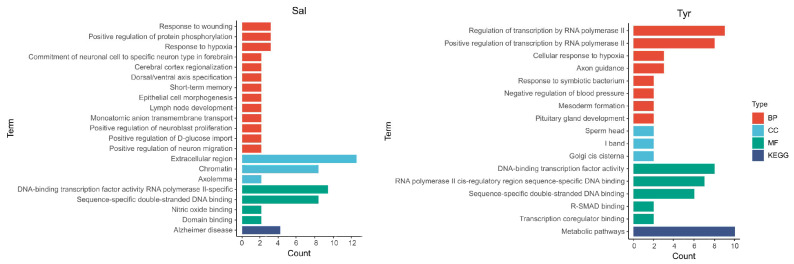
GO and KEGG pathway enrichment analysis of DEGs induced by salidroside and tyrosol. GO enrichment analysis. BP: Biological Process; CC: Cellular Component; MF: Molecular Function; KEGG: KEGG enrichment analysis. The x-axis represents the number of genes associated with each term, and the y-axis lists the enriched functional categories. Only significantly enriched terms are shown.

To systematically explore the biological processes and pathways associated with the transcriptional changes induced by salidroside and tyrosol, Gene Ontology (GO) enrichment analysis was performed using the Database for Annotation, Visualization and Integrated Discovery (DAVID) database. Enrichment analysis encompassed Biological Process (BP), Cellular Component (CC), Molecular Function (MF), and Kyoto Encyclopedia of Genes and Genomes (KEGG) pathway categories. A total of 21 significantly enriched GO terms were identified in salidroside-treated cells, whereas 17 enriched terms were identified following tyrosol treatment. The enriched GO terms and KEGG pathways are summarized and visualized as bar charts in Figure 3.

### 2.3. CMap Analysis

The up- and down-regulated DEGs induced by salidroside or tyrosol were used to query CMap. The output was exported as a ranked list of reference compounds with corresponding connectivity scores. Connectivity scores range from −1 to 1, where positive values indicate transcriptional similarity and negative values indicate opposing signatures relative to the query compound. Compounds with connectivity scores ≧ 0.8 or ≦−0.8 were considered strongly connected and are provided in Supplementary File S2.

For salidroside, a total of 69 compounds showed strong positive or negative connectivity, among which seven compounds were annotated to have targets related to the dopamine D2 receptor (DRD2). For tyrosol, 39 compounds exhibited strong connectivity, and eight of these compounds were associated with DRD2-related targets. The DRD2-targeting compounds identified from the CMap results are summarized in Table 1 (salidroside) and Table 2 (tyrosol).

Target annotations for all compounds were retrieved from the DrugBank database (https://go.drugbank.com). Only experimentally supported targets and mechanisms of action reported in previous studies were included for downstream interpretation [17].

### 2.4. Molecular Docking

Molecular docking was conducted to predict the binding modes of salidroside and tyrosol to DRD2 (Protein Data Bank (PDB) code: 8TQZ) and to estimate their binding affinities. Dopamine (an endogenous DRD2 agonist) and risperidone, sulpiride (DRD2 antagonists) were included as reference ligands.

**Figure 4:**
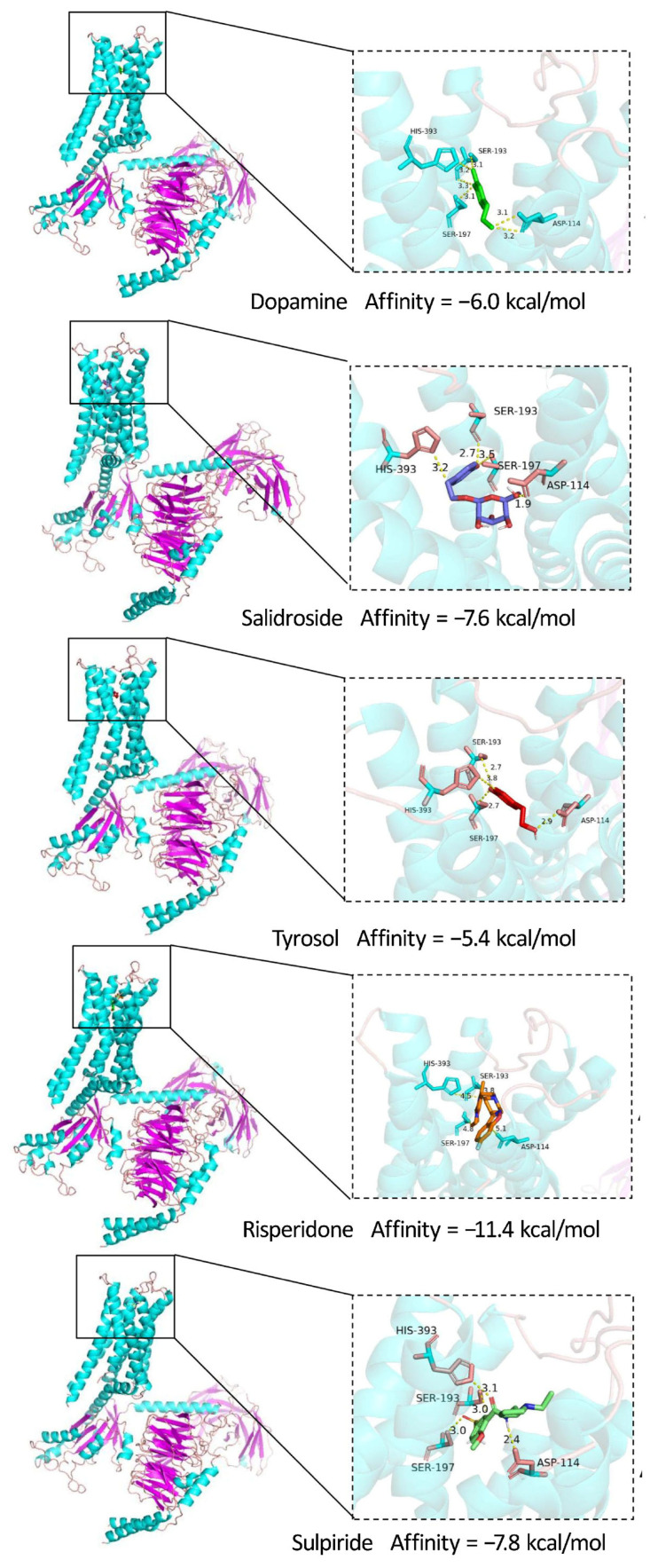
Molecular docking of dopamine, salidroside, tyrosol, risperidone and sulpiride with DRD2. Representative docking poses of dopamine, salidroside, tyrosol, risperidone and sulpiride within the predicted ligand-binding pocket of DRD2. Enlarged views highlight key interactions between each ligand and surrounding residues in the binding site.

The docking results indicated that salidroside and tyrosol occupied a binding pocket overlapping with that of dopamine, risperidone, and sulpiride (Figure 4), suggesting that these compounds may interact with DRD2 at a functionally relevant site. The predicted binding affinities (Vina scores) are summarized in Table 3. Notably, salidroside and tyrosol exhibited binding energies comparable to dopamine and sulpiride, supporting their potential to directly engage DRD2. All the investigated ligands form specific intermolecular interactions with the key amino acid residues Asp114, Ser193 and Ser197.

### 2.5. SPR Analysis

**Figure 5:**
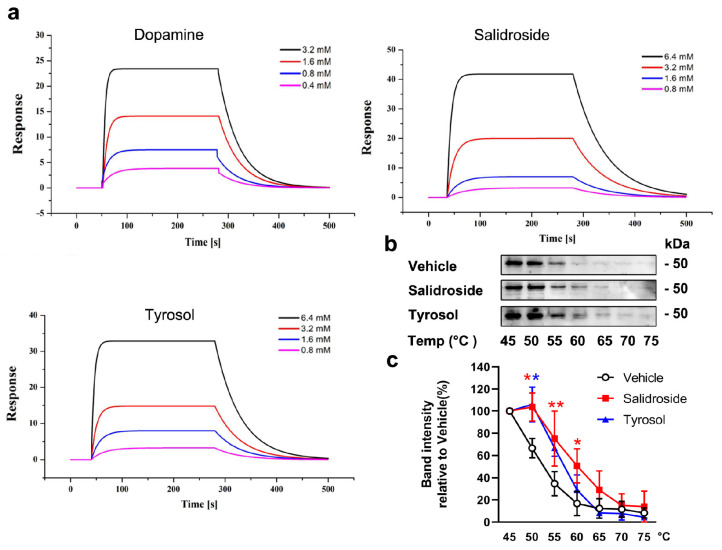
SPR analysis and CETSA of the interaction between DRD2 and dopamine, salidroside, or tyrosol. (**a**) Representative SPR sensorgrams showing concentration-dependent binding of dopamine, salidroside, and tyrosol to immobilized DRD2 (*n* = 3). (**b**) Representative Western blot analysis showing the thermal stability of DRD2 in A549 cells treated with vehicle, salidroside, or tyrosol. Cells were subjected to increasing temperatures, and the remaining soluble DRD2 protein in the supernatant was detected by immunoblotting. (**c**) Quantification of DRD2 protein levels (*n* = 3). Band intensities were normalized to the vehicle control and plotted as temperature-dependent thermal denaturation curves. A rightward shift in the curve indicates ligand-induced stabilization of DRD2 in the presence of salidroside or tyrosol. Results are presented as means ± standard error of the mean (SEM). Multiple group comparisons were assessed by one-way analysis of variance (ANOVA: F(2,42) = 5.377, *p* = 0.009), followed by the least significant difference (LSD) test for intergroup comparisons. * *p* < 0.05 and ** *p* < 0.01 vs. vehicle group.

SPR analysis was performed to evaluate the direct binding of salidroside and tyrosol to DRD2, with dopamine included as a reference ligand. Representative sensorgrams showing concentration-dependent binding of dopamine, salidroside, and tyrosol to immobilized DRD2 are presented in Figure 5a. The kinetic and affinity parameters derived from curve fitting are summarized in Table 4. The SPR results demonstrated that both salidroside and tyrosol bind directly to DRD2, exhibiting equilibrium dissociation constants (K_D_) in the high micromolar range. Although their binding affinities were lower than that of the endogenous ligand dopamine (K_D_ = 409 μM), both compounds showed measurable and reproducible interactions with DRD2, supporting their capacity for direct receptor engagement.

### 2.6. CETSA

CETSA was performed to examine the cellular target engagement of salidroside and tyrosol with DRD2. As temperature increased, DRD2 in the cell lysate progressively denatured and precipitated, resulting in a temperature-dependent decrease in soluble DRD2 detected in the supernatant. In comparison with the vehicle control, treatment with salidroside or tyrosol markedly enhanced the thermal stability of DRD2, as reflected by increased levels of soluble DRD2 remaining at elevated temperatures (*p* < 0.05 and *p* < 0.01) (Figure 5b). Quantitative analysis further revealed a rightward shift in the DRD2 thermal denaturation curve in the presence of salidroside or tyrosol (Figure 5c), indicating ligand-induced stabilization of DRD2 in cells.

### 2.7. Regulation of DRD2 Downstream Signaling by Salidroside and Tyrosol

#### 2.7.1. Effects of DRD2 Receptor Antagonism on Salidroside- and TyrosolInduced ERK Signaling

**Figure 6:**
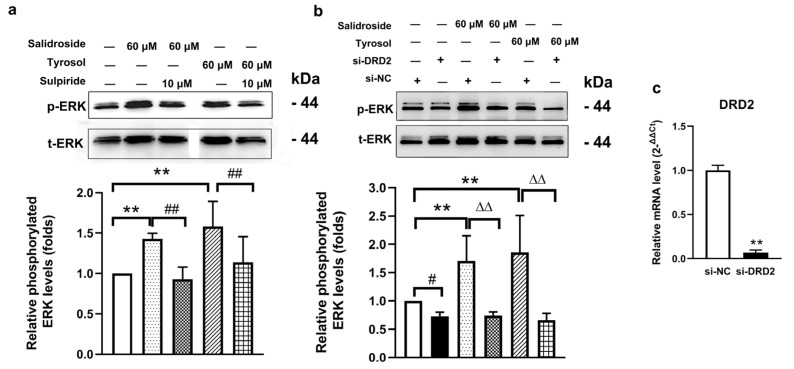
DRD2-dependent ERK activation induced by salidroside and tyrosol in SH-SY5Y cells. (**a**) Representative Western blot analysis and quantitative results showing ERK phosphorylation in SH-SY5Y cells treated with salidroside or tyrosol (60 μM) in the presence or absence of the DRD2 antagonist sulpiride (10 μM). p-ERK levels were normalized to t-ERK. Data are expressed as mean ± SEM (*n* = 5). Multiple group comparisons were assessed by one-way ANOVA: F(4,20) = 4.938, *p* = 0.0006, followed by LSD test for intergroup comparisons. ** *p* < 0.01, ^##^ *p* < 0.01. (**b**) Effects of DRD2 gene silencing on salidroside- and tyrosol-induced ERK phosphorylation. Cells were transfected with si-NC or si-DRD2 prior to compound treatment. Data are expressed as mean ± SEM (*n* = 9). Multiple group comparisons were assessed by one-way ANOVA (F(5,48) = 2.653, *p* = 0.033), followed by LSD test for intergroup comparisons. ** *p* < 0.01, ^#^ *p* < 0.05, ΔΔ *p* < 0.01. (**c**) Quantitative real-time PCR analysis of DRD2 mRNA levels confirming the efficiency of DRD2 knockdown. Data are expressed as mean ± SEM (*n* = 3). Intergroup comparison was performed with the independent samples *t*-test: t = 27.43, df = 4, *p* < 0.001. ** *p* < 0.01, compared vs. the si-NC group.

Treatment of SH-SY5Y cells with salidroside or tyrosol (60 μM) for 30 min significantly increased the phosphorylation level of ERK, a downstream signaling molecule of DRD2 (*p* < 0.01). However, pretreatment with the selective DRD2 antagonist sulpiride (10 μM) for 30 min completely abolished the salidroside- or tyrosol-induced increase in ERK phosphorylation (*p* < 0.01) (Figure 6a). Sulpiride alone did not significantly affect basal ERK phosphorylation levels. These results indicate that ERK activation induced by salidroside and tyrosol is dependent on functional DRD2 signaling.

#### 2.7.2. Effects of DRD2 Gene Silencing on Salidroside- and Tyrosol- Induced ERK Signaling

To further confirm the involvement of DRD2, RNA interference was used to silence DRD2 expression in SH-SY5Y cells. Quantitative real-time PCR analysis demonstrated that DRD2 mRNA levels were reduced to 6.75% of those observed in the control siRNA (si-NC) group (*p* < 0.01) (Figure 6c), confirming efficient gene silencing (Figure 6c). Consistent with the antagonist experiments, treatment with salidroside or tyrosol (60 μM, 30 min) markedly increased ERK phosphorylation in cells transfected with control siRNA (*p* < 0.01). In contrast, DRD2 knockdown completely abrogated ERK phosphorylation induced by salidroside or tyrosol (*p* < 0.01) (Figure 6c). These findings further support that ERK activation by salidroside and tyrosol requires the presence of DRD2.

## 3. Discussion

In the present study, we integrated transcriptomic profiling with CMap analysis and multi-level experimental validation to systematically identify the direct target of salidroside and its major in vivo metabolite, tyrosol. Using this strategy, we demonstrate that DRD2 represents a direct molecular target of both compounds. This conclusion is supported by convergent data from molecular docking, SPR, CETSA, and functional signaling analyses, thereby providing a mechanistic basis for the dopaminergic regulatory effects previously attributed to salidroside and tyrosol [4,13,18,19].

Identifying the direct molecular targets of bioactive compounds derived from medicinal plants remains a major challenge in pharmacological research. Currently, several methods have emerged, including labeled and non-labeled approaches [20]. Among them, CMap is a non-labeled method that works by linking input transcriptional “signatures” to gene expression profiles induced by thousands of small molecules with known mechanisms of action [14]. This strategy has been increasingly applied for drug repurposing, mechanism-of-action studies, and target discovery, particularly for compounds with unclear or multiple biological activities. In the context of traditional Chinese medicine and natural products, CMap offers a distinct advantage by enabling hypothesis generation without prior assumptions regarding chemical structure–target relationships [21]. To date, several studies have already applied this method for target discovery of compounds [22]. In this study, transcriptomic signatures derived from salidroside- and tyrosol-treated cells exhibited strong positive or negative connectivity with compounds known to modulate DRD2, highlighting the utility of CMap as an effective upstream screening tool for target identification. Importantly, CMap analysis alone does not establish causality; rather, it provides direction for subsequent experimental validation. Our study exemplifies how CMap-guided hypotheses can be rigorously tested and confirmed through complementary biochemical and cellular approaches.

A critical distinction in this study is the confirmation of direct physical binding. While previous studies have reported that salidroside modulates dopaminergic systems [23], it was unclear whether this was an indirect effect such as regulating synthesis enzymes or direct receptor interaction. Beyond target prediction, our study provides direct biochemical and biophysical evidence supporting the interaction between salidroside, tyrosol, and DRD2. Molecular docking analysis revealed that both compounds occupy a binding pocket overlapping with that of dopamine and the DRD2 antagonist risperidone [24], suggesting potential competition or functional relevance at the site. Importantly, our docking analyses revealed that salidroside and tyrosol share a conserved binding pattern with both DRD2 agonists and antagonists: similarly to the endogenous agonist dopamine, and the clinical antagonists risperidone and sulpiride, both compounds form stable hydrogen bonds with the core functional residues Asp114 and Ser193 of DRD2. It has been well established in prior work that Asp114 is the determinant residue for the high-affinity state (D2^High^R) of DRD2, whereas Ser193 is the key residue that mediates the low-affinity state (D2^Low^R) of the receptor [25]. Collectively, ligand engagement at these two conserved sites is a prerequisite for the biological activity and functional modulation of DRD2. SPR experiments further confirmed direct binding, demonstrating that salidroside and tyrosol interact with DRD2 with K_D_ values comparable to that of dopamine. Furthermore, CETSA analysis provided additional evidence, showing that both compounds increase the thermal stability of DRD2, indicating direct engagement with the receptor in a cellular context. These biophysical data provide strong evidence supporting DRD2 as a direct target of salidroside and tyrosol, rather than an indirect downstream effector.

Importantly, receptor binding was accompanied by functional signaling consequences. In SH-SY5Y cells, treatment with salidroside or tyrosol induced a significant increase in ERK phosphorylation, a well-established downstream signaling event of DRD2 activation [26,27]. This effect was abolished by pharmacological blockade with a DRD2 antagonist and by siRNA-mediated knockdown of DRD2, demonstrating that ERK activation is receptor-dependent. These findings indicate that salidroside and tyrosol engage DRD2 in a functionally relevant manner and activate downstream signaling pathways. Although the present data support agonist-like activation of DRD2-dependent ERK signaling, further studies are required to determine whether these compounds act as full agonists, partial agonists, or biased ligands with preferential pathway engagement.

The selection of different cell models in this study was guided by both methodological and biological considerations. A549 cells were used for transcriptomic profiling and CETSAs because they represent one of the core reference cell lines in the CMap database. Using the same cellular context as the CMap reference ensured that the transcriptional signatures generated by salidroside and tyrosol could be directly and reliably compared with existing perturbational profiles, thereby minimizing potential bias introduced by cell type-specific transcriptional programs. Consistent target engagement observed in A549 cells therefore provides a robust validation of the CMap-guided target prediction. In contrast, SH-SY5Y cells were employed for downstream functional signaling analyses due to their neuronal characteristics and well-established dopaminergic signaling machinery [28]. This complementary use of cell models allowed us to decouple target identification from functional consequence, while maintaining biological relevance for DRD2-mediated signaling.

It is noteworthy that the effect of salidroside on ERK signaling has been reported to differ across experimental systems. In several pathological models (e.g., inflammatory stimulation, hyperglycemic/oxidative stress conditions, and ischemia–reperfusion injury), salidroside has been reported to attenuate MAPK activation including reduced ERK1/2 phosphorylation [29,30]. In contrast, the ERK activation observed in the present study occurs rapidly and in a receptor-dependent manner, as evidenced by its sensitivity to DRD2 antagonism and gene silencing. This apparent discrepancy likely reflects context-dependent nature of ERK signaling [31], in which salidroside may attenuate pathological ERK overactivation under stress conditions while permitting or inducing transient ERK activation downstream of receptor-mediated signaling under physiological conditions. Such context-dependent regulation of ERK signaling has been widely observed and may contribute to the pleiotropic biological effects of neuromodulatory compounds across different experimental settings [32].

**Figure 7:**
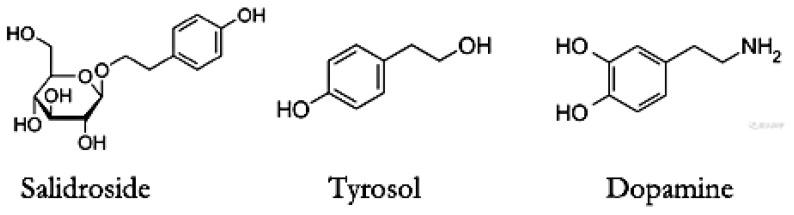
Chemical structures of salidroside, tyrosol, and dopamine. Salidroside is composed of a tyrosol aglycone conjugated to a glucose moiety. Tyrosol and dopamine share a phenethyl backbone, while dopamine additionally contains a catechol group and an ethylamine side chain.

The identification of DRD2 as a target of salidroside and tyrosol provides a mechanistic framework for understanding their previously reported effects on the dopaminergic system [13]. As a central regulator of dopaminergic neurotransmission, DRD2 functions as a key modulatory node integrating presynaptic and postsynaptic signaling, thereby fine-tuning dopamine release and downstream signal transduction [33]. Salidroside and tyrosol are phenylethanol derivatives, and tyrosol shares a phenethyl scaffold with dopamine and exhibits partial overlap in pharmacophoric features relevant to dopaminergic receptor recognition (as shown in Figure 7). Moreover, tyrosol participates in metabolic pathways closely related to dopamine metabolism. Tyrosol is metabolized by monoamine oxidase to 3,4-dihydroxyphenylacetaldehyde, or by aldehyde dehydrogenase to 3,4-dihydroxyphenylacetic acid, and then metabolized by catechol-O-methyl transferase to homovanillic acid; similarly, tyramine can be metabolized to tyrosol by monoamine oxidase and aldehyde/aldose reductase [34]. These biochemical relationships support the likelihood that tyrosol may interact with components of the dopaminergic system. In our previous studies, salidroside administration markedly increased dopamine levels and its metabolites in the striatum of cerebral ischemia–reperfusion model rats within a short time frame, suggesting a rapid regulatory effect on the dopaminergic system [13]. Accumulating evidence from previous studies also demonstrates that salidroside and tyrosol exert neuroprotective effects by acting on the dopaminergic neuronal system [4,18,19]. These observations provided an important rationale for prioritizing DRD2 as a candidate target following CMap screening. The present findings extend these observations by providing direct molecular evidence that DRD2 is a binding and signaling target of salidroside and tyrosol, thereby linking receptor-level engagement to system-level dopaminergic regulation.

Dopamine D2 receptor (DRD2) exerts a wide spectrum of physiological functions across multiple systems, including the regulation of neurotransmitter release, motor function modulation, affective and cognitive processing, endocrine homeostasis, and gastrointestinal function [35]. Among these functions, the regulatory role of DRD2 in neuromotor function has attracted extensive attention [36]. Reduced expression and functional dysregulation of DRD2 lead to motor symptoms including bradykinesia and tremor [37], a pathological phenotype that occurs not only in patients with Parkinson’s disease (PD), but also secondary to cerebral ischemia [38]. DRD2 agonists have been well documented to exert therapeutic effects on the aforementioned disorders [39,40]. G protein-coupled receptors (GPCRs) including DRD2 are promising future drug targets; a recent study characterizes the Gαₒ K46E mutation that locks G proteins in a pre-activated state, revealing the mechanism of related neurodevelopmental disorders and providing a novel tool for GPCR research and drug development [41]. Accordingly, the findings of our study provide a theoretical and experimental basis for the development of salidroside and tyrosol as potential dopaminergic neuroprotective agents.

Despite the strengths of this study, several limitations should be acknowledged. First, while CMap analysis proved valuable for hypothesis generation, it captures similarities in global transcriptional responses rather than direct pharmacological modalities at individual receptors [13,42]. For GPCR targets such as DRD2, compounds with distinct functional properties—including agonists, partial agonists, and antagonists—may elicit overlapping transcriptional signatures depending on cellular context, signaling bias, and exposure duration [43]. Therefore, the identification of both positively and negatively connected DRD2-modulating compounds in the CMap results is not contradictory, but instead highlights the complexity of downstream transcriptional regulation and underscores the necessity of complementary biochemical and functional assays to define the precise mode and directionality of receptor engagement.

A further limitation of our study relates to the SPR binding assays, in which we employed a commercially available recombinant human DRD2 protein (Abnova Corporation, Taipei City, Taiwan, Catalog No. H0001813-Q01). This protein comprises the partial open reading frame (ORF) of human DRD2 (AAH21195, amino acids 1-110), which encompasses the seven-transmembrane domains of the receptor. Critically, this recombinant protein presents in a low-affinity, G protein-uncoupled conformational state, which accounts for the discrepancy between the dopamine K_D_ value obtained in our assays and the well-characterized nanomolar K_D_ values reported in prior studies using membrane-based binding assays. In this context, it is important to clarify that the K_D_ values measured via SPR in our study are intended solely for the relative comparison of binding affinities across different ligands within the same experimental system, and not for the absolute quantification of physiologically relevant binding affinities under native cellular conditions.

To further delineate the signaling mechanisms and broader biological relevance of these interactions, several additional aspects warrant future investigation. First, dose–response analyses for salidroside and tyrosol, including determination of half maximal effective concentration (EC50) values for ERK activation are needed. Second, although ERK phosphorylation was used as a functional readout, additional signaling endpoints such as cyclic adenosine monophosphate (cAMP) modulation, β-arrestin recruitment, or G protein bias were not assessed. Moreover, DRD2 may not represent the sole target of salidroside and tyrosol, given their broad biological activities and the likelihood of multi-target engagement. In addition, the present findings were derived predominantly from cell models, and further in vivo investigations are necessary to clarify the physiological and therapeutic relevance of DRD2 modulation by these compounds.

## 4. Materials and Methods

### 4.1. Cell Culture and Drug Treatment

The A549 (American Type Culture Collection (ATCC) CCL-185) and SH-SY5Y (ATCC CRL-2266) cell lines were purchased from Dobiotech, Co. Ltd. (Daegu, Republic of Korea). The A549 cells were incubated in Dulbecco’s Modified Eagle Medium (DMEM) medium, and the SH-SY5Y cells were incubated in DMEM/F12 medium, both with 10% fetal bovine serum. All cell culture incubations were performed in a humidified 37 °C incubator with 5% CO_2_.

The A549 and SH-SY5Y cells were seeded into six-well plates at a density of 1.5 × 10^5^ cells per well 24 h before treatment. The next day, the medium was removed, and the cells were treated in a final volume of 2 mL.

### 4.2. RNA Sequencing and Analysis

For RNA sequencing, the A549 cells were seeded on six-well plates at a density of 1.5 × 10^5^ cells per well and cultured for 24 h prior to treatment. Then the medium was removed, 60 μM salidroside or tyrosol were added in a final volume of 2 mL when cell confluence reached approximately 50%. And Control cells received an equal volume of drug-free medium. Three independent biological replicates were set up for each group. After 12 h of treatment, the cells were collected using TRIzol (Life Technologies, Waltham, MA, USA) for total RNA extraction. RNA sequencing and primary data processing were performed by Shanghai Biotree Biotech (Shanghai, China). Briefly, RNA integrity was assessed using the RNA Nano 6000 Assay Kit on a Bioanalyzer 2100 system (Agilent Technologies, Santa Clara, CA, USA). For library preparation, 1 μg of total RNA per sample was used as the starting material, where messenger RNA (mRNA) was first purified from total RNA using poly-T oligo-attached magnetic beads and then fragmented with divalent cations at elevated temperature in 5× First Strand Synthesis Reaction Buffer. First-strand complementary DNA (cDNA) synthesis was performed using random hexamer primers and M-MuLV Reverse Transcriptase (RNase H), followed by second-strand synthesis using DNA Polymerase I and RNase H. The resulting cDNA fragments were subjected to end repair, 3′adenylation, and ligation with hairpin loop-structured adaptors.

cDNA fragments of 370–420 bp were selected using the AMPure XP system (Beckman Coulter, Brea, CA, USA), amplified by polymerase chain reaction (PCR) with Phusion High-Fidelity DNA polymerase, Universal PCR primers, and Index (X) Primer, and then purified (AMPure XP) before library quality assessment on the Agilent Bioanalyzer 2100. For clustering and sequencing, index-coded samples were clustered on a cBot system using TruSeq PE Cluster Kit v3-cBot-HS (Illumina, San Diego, CA, USA) according to the manufacturer’s protocol, followed by sequencing on an Illumina Novaseq platform to generate 150 bp paired-end reads.

Raw sequencing data were subjected to quality control to obtain clean reads. Reference genome and gene annotation files were downloaded from the corresponding genome database. Genome indexing was constructed using Hisat2 v2.0.5, and paired-end clean reads were aligned to the reference genome with the same tool. Reads mapped to each gene were counted using featureCounts v1.5.0-p3, and gene expression levels were normalized as fragments per kilobase of transcript per million mapped reads (FPKM).

Differential expression analysis was conducted using DESeq2 R package (version 1.20.0), which employs a negative binomial distribution-based model. *p* values were adjusted using the Benjamini–Hochberg method to control the false discovery rate (FDR). Genes with an adjusted *p* value < 0.05 were considered differentially expressed.

### 4.3. GO Enrichment Analysis

The DEG data identified from RNA sequencing analysis were submitted to the DAVID database for GO enrichment analysis (enriched terms were categorized into BP, CC, and MF), along with KEGG pathway analysis. Enrichment results were visualized using bar charts to display the number of DEGs associated with each functional category.

### 4.4. CMap Analysis

To identify potential molecular targets of salidroside and tyrosol, DEGs induced by each compound were queried against the CMap database(build 02) using the online platform provided by the Broad Institute (https://www.broadinstitute.org/cmap/, accessed on 1 February 2021). Genes exhibiting a fold change ≥ 1.5 (up-regulated or down-regulated relative to control) were included in the query signature, in accordance with the requirements of the CMap 2.0 analysis pipeline.

The resulting gene expression signatures were used to compute connectivity scores, which quantify the similarity between the transcriptional responses elicited by salidroside or tyrosol and those induced by reference compounds in the CMap database. Positive connectivity scores indicate similar transcriptional perturbations, whereas negative scores indicate opposing signatures. Compounds with connectivity scores greater than 0.8 or less than −0.8 were considered to exhibit strong positive or negative connectivity and were selected for further analysis.

The known molecular targets of the selected compounds were retrieved from the DrugBank database (https://go.drugbank.com) to facilitate target enrichment and mechanistic interpretation.

### 4.5. Molecular Docking

Molecular docking was performed using AutoDockTools (https://autodock.scripps.edu/, accessed on 7 March 2026), in combination with AutoDock Vina to predict the binding modes of dopamine, salidroside, tyrosol, risperidone and sulpiride to DRD2. The three-dimensional structures of the ligands were obtained from the PubChem database (https://pubchem.ncbi.nlm.nih.gov/). The crystal structure of DRD2 (PDB codes: 8TQZ) was retrieved from the Protein Data Bank (PDB).

Prior to docking, the receptor structure was prepared by removing non-essential molecules and adding appropriate hydrogen atoms. Redocking validation was performed with the co-crystallized dopamine ligand extracted from the DRD2 crystal structure, using preprocessing procedures and docking parameters identical to those applied to all other test ligands. A flexible docking approach was adopted, allowing the receptor and ligands to interact freely, and the corresponding grid energy map was generated. The docking results were evaluated based on the predicted binding poses and binding affinities (Vina scores). The best-ranked binding conformations were selected for visualization and analysis of ligand-receptor interactions.

### 4.6. SPR

SPR experiments were performed using a Nicoya OpenSPR™ instrument (Nicoya Lifesciences, Kitchener-Waterloo, ON, Canada) to quantitatively characterize the interactions between DRD2 and dopamine, salidroside, or tyrosol. A COOH sensor chip was activated according to the standard operating procedure provided by the OpenSPR™ system manufacturer. Recombinant DRD2 protein was immobilized onto the sensor surface at a concentration of 0.11 μg/μL by injecting 200 μL of protein solution into the sample loop over 4 min. Following immobilization, salidroside or tyrosol at serial concentrations (0.8, 1.6, 3.2, and 6.4 mM, prepared in phosphate-buffered saline (PBS)) was injected as analytes at a flow rate of 20 μL/min. Dopamine (0.4, 0.8, 1.6, and 3.2 mM) was included as a positive control. The association phase was set to 240 s, followed by a dissociation phase of 260 s under continuous buffer flow.

Sensorgrams obtained from concentration-dependent binding experiments were analyzed using TraceDrawer software V1.6.1 (Ridgeview Instruments AB, Uppsala, Sweden). Kinetic analysis was performed using a 1:1 Langmuir binding model. k_a_, k_d_, and K_D_ were calculated from the fitted curves.

### 4.7. Cellular Thermal Shift Assay (CETSA)

A549 cells were seeded in six-well plates and treated with salidroside or tyrosol (60 μM) for 0.5 h at 37 °C. To terminate the reaction, cells were washed with 300 μL of ice-cold PBS and harvested by scraping. The resulting cell suspensions were collected and subjected to a temperature gradient (45, 50, 55, 60, 65, 70, and 75 °C) for 3 min using a PCR thermal cycler (LongGene T10S, LongGene, Hangzhou, China). After heating, samples were immediately subjected to five freeze–thaw cycles between liquid nitrogen and room temperature to induce cell lysis. The lysates were then centrifuged at 12,000× *g* for 15 min at 4 °C to remove insoluble, thermally denatured proteins. The resulting supernatants, containing soluble protein fractions, were mixed with 5 × loading buffer and prepared for subsequent Western blot analysis as described in Section 4.10. Protein band intensities were quantified by densitometric analysis, and relative protein levels were normalized and plotted as temperature-dependent thermal stability curves.

### 4.8. Effects of DRD2 Receptor Antagonism on the Actions of Salidroside or Tyrosol

To examine whether the effects of salidroside and tyrosol on downstream signaling are mediated through DRD2, a pharmacological antagonism approach was employed. The human neuroblastoma cell line SH-SY5Y was used for these experiments. Phosphorylation of ERK, a downstream signaling molecule of DRD2, was assessed as a readout of receptor-mediated signaling activity.

SH-SY5Y cells were seeded into six-well plates at a density of 1.5 × 10^5^ cells per well and cultured for 24 h prior to treatment. Cells were pretreated with the DRD2 antagonist sulpiride (10 μM) for 30 min, followed by stimulation with salidroside or tyrosol (60 μM) for an additional 30 min. Control cells received vehicle treatment. After treatment, cells were washed with ice-cold PBS to terminate the reaction and lysed using protein lysis buffer. The lysates were centrifuged at 14,000× *g* for 10 min at 4 °C, and the supernatants were collected. Protein concentrations were determined using a BCA protein assay kit (Epizyme Biotech Co., Ltd., Shanghai, China) according to the manufacturer’s instructions. Loading buffer was added to the supernatants, and samples were denatured by heating at 95 °C for 5 min. The prepared samples were subsequently subjected to Western blot analysis.

### 4.9. Effects of DRD2 Receptor Gene Silencing on the Actions of Salidroside and Tyrosol

RNA interference (RNAi)-mediated knockdown of DRD2 was performed to further confirm the receptor dependence of salidroside- and tyrosol-induced signaling. SH-SY5Y cells were seeded into six-well plates at a density of 1.0 × 10^5^ cells per well and cultured for 24 h before transfection. Cells were transfected with DRD2-specific siRNA (DRD2-Homo-1113; Shanghai GenePharma Co., Ltd., Shanghai, China). The siRNA sequences were as follows: sense (5′-3′), CCGUUAUCAUGAAGUCUAATT; antisense (3′-5′), UUAGACUUCAUGAUAACGGTT. For transfection, 1 nM siRNA and 5 μL of Entraster™-R4000 transfection reagent (Engreen Biosystem, Ltd., Auckland, New Zealand) were mixed with 250 μL of serum-free DMEM/F12 medium and incubated for 15 min at room temperature. The transfection mixture was then added to each well and incubated with the cells for 24 h.

Following transfection, cells were treated with salidroside or tyrosol (60 μM) for 30 min. Cells were subsequently harvested, and total protein was extracted for Western blot analysis as described in Section 4.10. In parallel, a separate set of cells was collected for total RNA extraction using TRIzol reagent, and the efficiency of DRD2 knockdown was evaluated by quantitative real-time PCR.

### 4.10. Western Blot Analysis

Western blot analysis was performed according to standard procedures. For CETSA experiments, 50 μL of each sample was loaded per lane, whereas for receptor antagonism experiments, 30 μg of total protein was loaded per lane. Proteins were separated by sodium dodecyl sulfate-polyacrylamide gel electrophoresis (SDS-PAGE) and subsequently transferred onto the 0.45 μm polyvinylidene fluoride (PVDF) membrane. Membranes were blocked with 5% bovine serum albumin (BSA) in Tris-buffered saline with Tween 20 (TBST) for 2 h in room temperature, followed by incubation with the primary antibodies overnight at 4 °C. The primary antibodies used were as follows: a goat anti-DRD2 antibody (1:1000, ab30743, Abcam, Cambridge, UK) and a rabbit anti-phospho-ERK antibody (1:1000, CST #4370, Cell Signaling Technology, Danvers, MA, USA). After washing three times in 1×TBST, membranes were incubated with horseradish peroxidase (HRP)-conjugated rabbit anti-goat secondary antibody (1:2000, BA1006, Boster, Wuhan, China) at room temperature for 2 h. Protein bands were visualized using enhanced chemiluminescence (ECL) reagents after appropriate washing steps. For total ERK blotting, the membranes were stripped using stripping buffer and re-incubated overnight at 4 °C with ERK primary antibody (1:2000, Abclonal A4782, Abclonal Technology, Wuhan, China) overnight at 4 °C, followed by incubation with the corresponding secondary antibody and ECL detection. Finally, images were acquired using a FluorChem M System (ProteinSimple, Santa Clara, CA, USA). Band intensities were quantified using ImageJ software 1.54 (LOCI, University of Wisconsin, Madison, WI, USA).

### 4.11. Real-Time PCR Assay

Total RNA was extracted using TRIzol reagent according to the manufacturer’s instructions. RNA concentration and purity were determined in an ultra-micro-spectrophotometer (Nanodrop2000, Thermo, Waltham, MA, USA). Afterwards, reverse transcription was performed using a First Strand cDNA Synthesis Kit (F. Hoffmann-La Roche Ltd., South San Francisco, CA, USA). Quantitative real-time PCR was carried out using an ABI StepOne plus real-time PCR system (Applied Biosystems, Carlsbad, CA, USA) with the FastStart Universal SYBR Green Master Mix (With ROX) (F. Hoffmann-La Roche Ltd., CA, USA). The amplification procedure was as follows: predenaturation at 95 °C for 10 min (95 °C, 15 s→60 °C, 60 s) × 40 cycles. The melting curve was measured at 60 °C→95 °C with a temperature increment of 0.3 °C per 15 s. The relative target gene expression was calculated using the 2^−ΔΔCT^ method. Glyceraldehyde-3-phosphate dehydrogenase (GAPDH) was used as the internal reference gene. All primers were synthesized by Servicebio (Wuhan, China). The primer used were as follows: HOMO-DRD2 forward primer (5′-3′): GGTAATGCCGTGGGTTGTCT; HOMO-DRD2 reverse primer (5′-3′): TTGTTGAGTCCGAAGAGCAGT; HOMO-GAPDH forward primer (5′-3′): GGAAGCTTGTCATCAATGGAAATC; HOMO-GAPDH reverse primer (5′-3′): TGATGACCCTTTTGGCTCCC.

### 4.12. Statistical Analysis

All data are presented as mean ± standard error of the mean (SEM). For normally distributed data, statistical analyses were performed using one-way analysis of variance (ANOVA). Post hoc intergroup comparisons were conducted using the least significant difference (LSD) test when variances were homogeneous, or the Games–Howell test when variances were unequal. A two-tailed *p*-value < 0.05 was considered statistically significant. All analyses were performed using GraphPad Prism 8 software.

## 5. Conclusions

In conclusion, this study identifies DRD2 as a direct molecular target of salidroside and tyrosol through an integrated strategy combining transcriptomics, CMap analysis, and rigorous experimental validation. These findings provide mechanistic insight into the dopaminergic regulatory effects of salidroside and tyrosol and highlight the value of CMap-guided target discovery for natural products. Our work lays a foundation for future investigations into the context-dependent roles of DRD2 modulation in the neuroprotective and neuropharmacological actions of salidroside and tyrosol, and support the potential of these compounds as lead molecules for the modulation of dopaminergic signaling pathways.

## Figures and Tables

**Table 1:** DRD2-related compounds identified by Connectivity Map analysis of salidroside-induced gene expression signatures.

Score	Drug Name	Target and Action
0.901	Clozapine	Antagonist of DRD2/HTR2A
0.808	Domperidone	Antagonist of DRD3/DRD2
−0.836	Haloperidol	Antagonist of DRD2; associates with HTR2C
−0.848	Perphenazine	Antagonist of DRD2/DRD1
−0.87	Levodopa	Agonist of DRD1/DRD5/DRD2/DRD3/DRD4
−0.923	Acepromazine	Antagonist of DRD2/DRD1/HTR2A/HTR1A
−0.99	Amantadine	Inhibitor of Matrix protein 2; antagonist of NMDAR 3A/DRD2

Footnotes: Connectivity scores were obtained from CMap (build 02) analysis using differentially expressed genes (fold change ≧ 1.5) induced by salidroside treatment. Positive scores indicate transcriptional similarity, whereas negative scores indicate opposing signatures relative to salidroside. Only compounds with experimentally validated targets annotated in the DrugBank database were included. Target annotations represent the primary known molecular targets and pharmacological actions reported in previous studies. DRD: dopamine receptor; HTR: 5-hydroxytryptamine receptor; NMDAR: N-methyl-D-aspartate receptor.

**Table 2:** DRD2-related compounds identified by Connectivity Map analysis of tyrosol-induced gene expression signatures.

Score	Drug Name	Target and Action
0.825	Prochlorperazine	Antagonist of DRD2
−0.818	Haloperidol	Antagonist of DRD2; associates with HTR2C
−0.821	Promazine	Antagonist of DRD2
−0.826	Chlorpromazine	Antagonist of DRD2/HTR1A/HTR2A/ADRA1A/ADRA1B/HRH1
−0.883	Fluphenazine	Antagonist of DRD2/DRD1
−0.895	Trifluoperazine	Antagonist of DRD2/CALY/ADRA1A
−0.941	Sulpiride	Antagonist of DRD2
−0.974	Thioridazine	Antagonist of DRD2/DRD1/ADRA1A/HTR2A

Footnotes: Connectivity scores were derived from CMap (build 02) analysis using differentially expressed genes (fold change ≧ 1.5) regulated by tyrosol treatment. Compounds with connectivity scores > 0.8 or <−0.8 were considered strongly connected. Positive scores indicate similar transcriptional responses, whereas negative scores indicate opposing signatures. Target information was retrieved from the DrugBank database and limited to experimentally supported targets. HR: histamine receptor; ADR: adrenaline receptor.

**Table 3:** Predicted binding affinities of dopamine, salidroside, tyrosol, risperidone and sulpiride to DRD2 obtained from molecular docking analysis.

		Center			Size		
Molecule	Affinity (kcal/mol)	x	y	z	x	y	z
Dopamine	−6.0	153	136	132	27	30	35
Salidroside	−7.6	153	135	132	22	30	35
Tyrosol	−5.4	165	167	190	25	17	17
Rispedone	−11.1	165	167	190	26	26	26
Sulpiride	−7.8	165	167	190	22	22	22

Footnotes: Molecular docking was performed using AutoDock Vina. Binding affinities are reported as Vina scores (kcal/mol), with more negative values indicating stronger predicted binding. Dopamine (endogenous agonist) and risperidone, sulpiride (antagonist) were included as reference ligands. Docking scores represent predicted binding energies and do not directly reflect experimental binding affinities.

**Table 4:** Kinetic and affinity parameters for the interaction between DRD2 and dopamine, salidroside, or tyrosol determined by SPR analysis.

	Parameters	Dopamine	Salidroside	Tyrosol
Association Rate Constant	k_a_ (1/(M·s))	60.8	17.6	25.3
Dissociation Rate Constant	k_d_ (1/s)	2.48 × 10^−2^	1.66 × 10^−2^	2.05 × 10^−2^
Dissociation Equilibrium Constant	K_D_ (μM)	409	943	813

Footnotes: Kinetic parameters, including the association rate constant (kₐ), dissociation rate constant (k_d_), and equilibrium dissociation constant (K_D_), were obtained by fitting SPR sensorgrams using a 1:1 Langmuir binding model. Dopamine was included as a reference ligand. K_D_ values represent equilibrium dissociation constants derived from kinetic analysis.

## Data Availability

The original contributions presented in this study are included in the article/Supplementary Material. Further inquiries can be directed to the corresponding authors.

## Supplementary Materials

The following supporting information can be downloaded at: https://www.mdpi.com/article/10.3390/ph19040540/s1, Supplementary File S1: Differentially expressed genes after treatment by salidroside (*p* < 0.05). Supplementary File S2: Compounds with connectivity scores ≧ 0.8 or ≦ −0.8 after treatment by salidroside.
