# Supplementary material for: Identification of Dopamine D2 Receptor as a Direct Target of Salidroside and Tyrosol by Integrated Transcriptomic and Biophysical Approaches
Source: Pharmaceuticals (Basel). 2026 Mar 27;19(4):540. doi: 10.3390/ph19040540 (PMC13119093; doi:10.3390/ph19040540)
Supplement: Supplementary file 1 [file pharmaceuticals-19-00540-s001.zip › Supplementary File S2.pdf]

Supplementary File S2

Compounds with connectivity scores ≧ 0.8 or ≦ −0.8 after treatment by salidroside.

Rank	CMAP Name	Dose	Cell	Score
1	Mefloquine	10 µM	MCF7	1
2	Seneciphylline	12 µM	HL60	1
3	Securinine	18 µM	MCF7	0.984
4	Disulfiram	13 µM	MCF7	0.956
5	Pioglitazone	10 µM	PC3	0.937
6	Iobenguane	11 µM	MCF7	0.926
7	15-Delta Prostaglandin J2	10 µM	MCF7	0.919
8	Dextromethorphan	11 µM	MCF7	0.918
9	Securinine	18 µM	MCF7	0.909
**10**	**Clozapine**	**12 µM**	**HL60**	**0.901**
11	Dorzolamide	11 µM	HL60	0.898
12	Parthenolide	16 µM	MCF7	0.894
13	Valproic Acid	1 mM	HL60	0.887
14	15-Delta Prostaglandin J2	10 µM	MCF7	0.886
15	Nocodazole	13 µM	PC3	0.884
16	Monorden	100 nM	MCF7	0.881
17	Loperamide	8 µM	PC3	0.88
18	Natamycin	6 µM	MCF7	0.875
19	Flecainide	8 µM	MCF7	0.868
**20**	**Phenelzine**	**17 µM**	**PC3**	**0.863**
21	Parthenolide	16 µM	PC3	0.863
22	Tanespimycin	1 µM	HL60	0.861
23	Prestwick-642	14 µM	PC3	0.858
24	Mifepristone	9 µM	MCF7	0.852
25	Dydrogesterone	13 µM	HL60	0.849
26	Geldanamycin	1 µM	PC3	0.846
27	Ciclacillin	12 µM	MCF7	0.843
28	Phenoxybenzamine	12 µM	MCF7	0.841
29	Dexibuprofen	19 µM	HL60	0.841
30	Withaferin A	1 µM	MCF7	0.836
31	Disulfiram	13 µM	PC3	0.835
32	Prestwick-864	35 µM	PC3	0.833
33	Carcinine	22 µM	MCF7	0.831
34	Pregnenolone	13 µM	HL60	0.83
35	Trifluridine	14 µM	MCF7	0.83
36	Corticosterone	12 µM	MCF7	0.829
37	Clioquinol	13 µM	MCF7	0.827
38	Withaferin A	1 µM	MCF7	0.827
39	Dihydrostreptomycin	3 µM	HL60	0.825

40	Valproic Acid	1 mM	HL60	0.82
41	Proguanil	14 µM	MCF7	0.818
42	Novobiocin	6 µM	HL60	0.818
43	Sulfadimidine	13 µM	MCF7	0.813
44	Monorden	100 nM	MCF7	0.812
45	Carmustine	100 µM	MCF7	0.812
**46**	**Domperidone**	**7 µM**	**HL60**	**0.808**
47	Diazoxide	17 µM	PC3	0.807
48	15-Delta Prostaglandin J2	10 µM	HL60	0.804
49	Naringenin	15 µM	HL60	0.804
50	Monorden	100 nM	HL60	0.804
51	Ritodrine	12 µM	HL60	0.804
52	F0447-0125	10 µM	MCF7	0.804
53	Oxaprozin	14 µM	PC3	0.801
54	Metolazone	11 µM	MCF7	0.801
6050	Calcium Pantothenate	8 µM	HL60	-0.804
6051	Nitrofurantoin	17 µM	HL60	-0.807
6052	Pilocarpine	15 µM	HL60	-0.808
6053	Aminophenazone	17 µM	HL60	-0.809
6054	Lobelanidine	11 µM	MCF7	-0.812
6055	Azathioprine	14 µM	HL60	-0.814
6056	Procainamide	15 µM	MCF7	-0.816
6057	Neomycin	4 µM	MCF7	-0.818
6058	Acetazolamide	18 µM	HL60	-0.821
6059	LY-294002	10 µM	HL60	-0.823
6060	Novobiocin	6 µM	MCF7	-0.824
6061	PHA-00851261E	10 µM	PC3	-0.825
6062	Isoniazid	29 µM	PC3	-0.825
6063	Nitrendipine	11 µM	MCF7	-0.826
6064	Fenbufen	16 µM	MCF7	-0.827
6065	0173570-0000	10 µM	PC3	-0.827
6066	Vincamine	11 µM	PC3	-0.828
6067	Dacarbazine	22 µM	HL60	-0.831
6068	BCB000040	10 µM	PC3	-0.832
6069	AG-013608	10 µM	MCF7	-0.834
6070	Stachydrine	22 µM	HL60	-0.835
**6071**	**Haloperidol**	**10 µM**	**HL60**	**-0.836**
6072	Oleandomycin	5 µM	HL60	-0.836
6073	Zalcitabine	19 µM	HL60	-0.838
6074	CP-645525-01	10 µM	PC3	-0.839
6075	Xylazine	18 µM	PC3	-0.84
6076	Cefalexin	11 µM	HL60	-0.841
6077	Metrifonate	16 µM	HL60	-0.845
6078	Perphenazine	10 µM	HL60	-0.848

6079	Zimeldine	10 µM	HL60	-0.848
6080	Tracazolate	12 µM	MCF7	-0.851
6081	PNU-0230031	10 µM	PC3	-0.851
6082	Ketoconazole	8 µM	HL60	-0.852
6083	Prestwick-665	12 µM	PC3	-0.853
6084	Prednisolone	11 µM	HL60	-0.858
6085	Methoxamine	16 µM	HL60	-0.859
6086	Iproniazid	14 µM	MCF7	-0.87
**6087**	**Levodopa**	**20 µM**	**HL60**	**-0.87**
6088	Nilutamide	13 µM	MCF7	-0.879
6089	Levobunolol	12 µM	HL60	-0.885
6090	Tribenoside	8 µM	PC3	-0.886
6091	Moxonidine	17 µM	HL60	-0.892
6092	Ethosuximide	28 µM	HL60	-0.896
6093	Terbutaline	7 µM	PC3	-0.914
**6094**	**Acepromazine**	**9 µM**	**HL60**	**-0.923**
6095	Prestwick-857	12 µM	MCF7	-0.93
6096	Phensuximide	21 µM	MCF7	-0.943
6097	Propranolol	14 µM	PC3	-0.96
6098	Metamizole Sodium	12 µM	MCF7	-0.972
**6099**	**Amantadine**	**10 µM**	**PC3**	**-0.99**
6100	Bethanechol	20 µM	HL60	-1

2. Compounds with connectivity scores ≧ 0.8 or ≦ −0.8 after treatment by tyrosol.

Rank	CMAP Name	Dose	Cell	Score
1	Levopropoxyphene	7 µM	HL60	1
2	Metronidazole	23 µM	PC3	0.931
3	Merbromin	5 µM	HL60	0.928
4	Diethylstilbestrol	15 µM	HL60	0.901
5	Estradiol	100 nM	MCF7	0.9
6	Cyclic Adenosine Monophosphate	12 µM	HL60	0.895
7	Nadolol	13 µM	HL60	0.862
8	Benzathine Benzylpenicillin	4 µM	PC3	0.845
9	Estradiol	15 µM	HL60	0.845
10	Flucloxacillin	8 µM	HL60	0.836
11	Cefepime	7 µM	PC3	0.836
12	Tropine	28 µM	PC3	0.834
**13**	**Prochlorperazine**	**7 µM**	**HL60**	**0.825**
14	Chlorcyclizine	12 µM	HL60	0.825
15	Moracizine	9 µM	HL60	0.807
16	Lansoprazole	11 µM	HL60	0.802
6006	Cyclopenthiazide	11 µM	PC3	-0.8

6007	Bromocriptine	5 µM	HL60	-0.8
6008	Fluorocurarine	12 µM	HL60	-0.8
6009	Mebendazole	14 µM	HL60	-0.801
6010	Pronetalol	15 µM	HL60	-0.802
6011	Metamizole Sodium	12 µM	PC3	-0.802
6012	Profenamine	11 µM	HL60	-0.803
6013	Meclozine	9 µM	HL60	-0.803
6014	Ipratropium Bromide	10 µM	HL60	-0.803
6015	Bemegride	26 µM	PC3	-0.805
6016	Prestwick-685	11 µM	PC3	-0.805
6017	Etofylline	18 µM	PC3	-0.806
6018	Luteolin	14 µM	HL60	-0.806
6019	Cloperastine	11 µM	HL60	-0.806
6020	BCB000040	10 µM	MCF7	-0.81
6021	0317956-0000	10 µM	MCF7	-0.811
6022	Rimexolone	11 µM	MCF7	-0.812
6023	Nicotinic Acid	32 µM	HL60	-0.814
6024	Urapidil	9 µM	HL60	-0.815
6025	Canavanine	14 µM	MCF7	-0.817
6026	Sertaconazole	8 µM	PC3	-0.818
6027	Fluspirilene	8 µM	HL60	-0.818
6028	Sirolimus	100 nM	MCF7	-0.818
**6029**	**Haloperidol**	**10 µM**	**HL60**	**-0.818**
6030	Sirolimus	100 nM	MCF7	-0.819
6031	Succinylsulfathiazole	11 µM	HL60	-0.819
**6032**	**Promazine**	**12 µM**	**PC3**	**-0.821**
6033	LY-294002	10 µM	MCF7	-0.821
6034	Trolox C	16 µM	MCF7	-0.822
6035	Skimmianine	15 µM	HL60	-0.822
6036	Acetylsalicylic Acid	100 µM	HL60	-0.823
6037	LY-294002	10 µM	PC3	-0.824
6038	Piribedil	12 µM	HL60	-0.824
6039	Ethotoin	20 µM	HL60	-0.825
6040	Cantharidin	20 µM	HL60	-0.825
**6041**	**Chlorpromazine**	**1 µM**	**HL60**	**-0.826**
6042	Chloramphenicol	12 µM	HL60	-0.829
6043	Zimeldine	10 µM	PC3	-0.834
6044	Prazosin	10 µM	HL60	-0.835
6045	Wortmannin	10 nM	PC3	-0.835
6046	Tyloxapol	4 µM	HL60	-0.836
6047	Cefaclor	10 µM	HL60	-0.837
6048	0175029-0000	1 µM	PC3	-0.839
6049	Pentamidine	7 µM	HL60	-0.839
6050	Cefalexin	11 µM	HL60	-0.841

6051	Aminohippuric Acid	21 µM	HL60	-0.841
6052	Fusaric Acid	22 µM	HL60	-0.842
6053	Fulvestrant	1 µM	MCF7	-0.842
6054	Indapamide	11 µM	PC3	-0.842
6055	Sulfaphenazole	13 µM	HL60	-0.843
6056	Nilutamide	13 µM	MCF7	-0.844
6057	Norfloxacin	13 µM	PC3	-0.846
6058	Flunixin	8 µM	HL60	-0.846
6059	Pipemidic Acid	13 µM	HL60	-0.848
6060	Ethotoin	20 µM	PC3	-0.849
6061	Tolbutamide	15 µM	PC3	-0.85
6062	Meptazinol	15 µM	HL60	-0.852
6063	Fursultiamine	9 µM	HL60	-0.855
6064	Naringin	7 µM	HL60	-0.859
6065	Napelline	11 µM	HL60	-0.859
6066	Sirolimus	100 nM	PC3	-0.859
6067	Tolazamide	13 µM	HL60	-0.86
6068	Pivampicillin	9 µM	HL60	-0.861
6069	Amylocaine	15 µM	PC3	-0.863
6070	Tolbutamide	15 µM	HL60	-0.863
6071	Quinostatin	10 µM	MCF7	-0.864
6072	Sulfadimidine	13 µM	HL60	-0.871
6073	Etamsylate	15 µM	HL60	-0.872
6074	Epirizole	17 µM	PC3	-0.874
6075	Fluvoxamine	9 µM	HL60	-0.879
6076	Rescinnamine	6 µM	MCF7	-0.88
6077	Gossypol	8 µM	HL60	-0.882
**6078**	**Fluphenazine**	**10 µM**	**PC3**	**-0.883**
6079	Tretinoin	1 µM	HL60	-0.889
6080	Trifluoperazine	8 µM	HL60	-0.89
6081	Metyrapone	18 µM	HL60	-0.891
**6082**	**Trifluoperazine**	**10 µM**	**HL60**	**-0.895**
6083	Picrotoxinin	14 µM	HL60	-0.9
6084	Terguride	9 µM	HL60	-0.9
6085	Pempidine	13 µM	MCF7	-0.901
6086	Zalcitabine	19 µM	HL60	-0.906
6087	Labetalol	11 µM	HL60	-0.909
6088	Lymecycline	7 µM	HL60	-0.91
6089	Flavoxate	9 µM	MCF7	-0.911
6090	Memantine	19 µM	HL60	-0.911
6091	Wortmannin	10 nM	MCF7	-0.918
6092	Sulfadiazine	16 µM	PC3	-0.921
**6093**	**Sulpiride**	**12 µM**	**PC3**	**-0.941**
6094	Cefalotin	10 µM	HL60	-0.945

6095	Mephentermine	9 µM	HL60	-0.952
6096	Sirolimus	100 nM	PC3	-0.954
6097	Naftopidil	9 µM	HL60	-0.958
6098	LY-294002	10 µM	MCF7	-0.968
**6099**	**Thioridazine**	**10 µM**	**HL60**	**-0.974**
6100	Iopanoic Acid	7 µM	HL60	-1
