# Supplementary material for: Identification of Dopamine D2 Receptor as a Direct Target of Salidroside and Tyrosol by Integrated Transcriptomic and Biophysical Approaches
Source: Pharmaceuticals (Basel). 2026 Mar 27;19(4):540. doi: 10.3390/ph19040540 (PMC13119093; doi:10.3390/ph19040540)
Supplement: Supplementary file 1 [file pharmaceuticals-19-00540-s001.zip › Supplementary File S1.pdf]

Supplementary File S1

Differentially expressed genes after treatment by salidroside ($p < 0.05$)

Gene name	Log2 Fold change	Gene name	Log2 Fold change	Gene name	Log2 Fold change
INSL6	4.80	CTBS	0.57	FAM169A	0.41
CBSL	4.53	GCSH	0.54	RWDD1	0.40
ABCA13	4.43	HECA	0.54	MAP9	0.40
FBXO47	4.29	EIF2S3B	0.53	ERGIC2	0.40
MPP4	4.18	NALCN	0.52	SHPRH	0.40
TLX1	4.05	XRCC4	0.52	ELL2	0.40
SIT1	4.00	CAPS2	0.52	ZNF322	0.40
ZFR2	3.58	COMMD6	0.51	CARNMT1	0.40
KCNT2	3.21	RAPH1	0.50	CCDC18	0.39
BARHL1	2.70	SAMD9	0.50	PTPN4	0.39
UBE2Q2L	2.42	LDHB	0.50	ZEB1	0.39
U2AF1	2.26	MANEA	0.50	POLE2	0.39
TUBB8	2.05	ZNF254	0.49	OSBPL8	0.39
ZNF474	1.98	NDUFC2	0.49	WDR72	0.39
UGT1A6	1.97	CHRFAM7A	0.49	LEPR	0.39
PIWIL4	1.93	ANKRD18A	0.49	C18orf54	0.39
SPANXB1	1.31	NBPF20	0.47	ERCC6L2	0.39
RGPD5	1.25	INTS12	0.45	KIF20B	0.39
RCBTB2	1.16	PIBF1	0.45	MOB4	0.39
CFAP46	1.16	CENPE	0.45	ABHD13	0.38
GUCY1B1	1.12	PDE3B	0.45	ANKRD12	0.38
C12orf60	0.89	FAM171B	0.45	NBEAL1	0.38
LMO3	0.82	RAB3IP	0.44	C12orf4	0.38
CCDC126	0.82	CXCL8	0.43	TROVE2	0.38
EDDM13	0.74	RBBP8	0.43	REV3L	0.38
SYCP2	0.70	GSTCD	0.43	CEP290	0.38
ANKRD18B	0.68	ZNF529	0.42	ZNF92	0.38
SRSF12	0.66	TRIP11	0.42	USP34	0.38
GPR89B	0.66	ZBTB10	0.42	RPGRIP1L	0.38
SERTAD4	0.65	TMTC3	0.42	INTS6	0.38
CFAP300	0.64	LIN7A	0.41	RBM26	0.38
CD2AP	0.38	BAZ2B	0.34	ZNF770	0.33
AGL	0.37	ZDHHC21	0.34	CCDC88A	0.33
ARHGAP5	0.37	TMEM67	0.34	SELENOI	0.33
UFL1	0.37	ZFYVE16	0.34	FANCM	0.32
BCAP29	0.37	HSD17B12	0.34	ASPM	0.32
FAM135A	0.37	HIF1A	0.34	AKAP11	0.32

MYBL1	0.37	SLC36A4	0.34	NEBL	0.32
CEP135	0.37	EPC2	0.34	PTAR1	0.32
JMJD1C	0.37	PON3	0.34	ATAD2	0.32
IKBIP	0.37	BRCA1	0.34	BIRC6	0.32
PARPBP	0.37	EIF5A2	0.34	DYNC2H1	0.32
LTN1	0.37	SRBD1	0.34	SETX	0.32
ZNF267	0.36	SLC4A7	0.34	CCPG1	0.32
ROCK1	0.36	CCDC91	0.34	PUS7L	0.32
PRPF40A	0.36	OGFRL1	0.34	SMCHD1	0.32
STXBP4	0.36	LPP	0.34	EFR3A	0.32
CCDC144A	0.36	PRRG1	0.34	TMED5	0.32
JAK2	0.36	C1GALT1	0.34	FAM92A	0.32
TBPL1	0.36	ARMC1	0.34	MAP4K5	0.32
EDRF1	0.36	PPP3CA	0.34	SCAF11	0.32
SMC6	0.36	USP45	0.34	ZMYM1	0.32
FANCB	0.36	FNIP1	0.34	SLK	0.32
CCNC	0.35	TMEM56	0.33	TAB2	0.32
USP37	0.35	MAP2K6	0.33	SNX14	0.32
SPAG1	0.35	STAG2	0.33	RB1	0.32
ZCCHC10	0.35	TENT4B	0.33	DNAH14	0.32
NBN	0.35	PDS5B	0.33	ASCC3	0.31
HMMR	0.35	KIAA1109	0.33	FBXO30	0.31
CRYBG3	0.35	FAM111B	0.33	SACS	0.31
SMC2	0.35	FAM208B	0.33	RIF1	0.31
IL6ST	0.35	POLK	0.33	ZNF480	0.31
KLHL28	0.35	FASTKD2	0.33	ANLN	0.31
SGO2	0.35	PIK3C2A	0.33	KIAA1551	0.31
QSER1	0.35	ZNF451	0.33	STAG1	0.31
CHML	0.35	WWP1	0.33	TEX10	0.31
CHD9	0.35	CEP57	0.33	SMARCAD1	0.31
PHIP	0.35	SUV39H2	0.33	POLR3G	0.31
SLC41A2	0.35	TMEM106B	0.33	MIS18BP1	0.31
CDK6	0.35	CEP152	0.33	ROCK2	0.31
MAP1B	0.35	VPS13C	0.33	PLEKHA3	0.31
B4GALT6	0.35	RUFY2	0.33	CLOCK	0.31
LIN54	0.35	SSX2IP	0.33	DNTTIP2	0.31
TMEM170B	0.35	GOLGA4	0.33	AC011043.1	0.31
WASHC4	0.34	PLCE1	0.33	TM4SF18	0.31
SHTN1	0.31	CWC22	0.29	FAM126A	0.28
RAD18	0.31	LRRC58	0.29	PPP1R12A	0.28
BROX	0.31	RALGAPA1	0.29	PKN2	0.28
GTF2A1	0.31	ZYG11B	0.29	MAPK6	0.28
ZNF655	0.31	VPS8	0.29	KTN1	0.28
MGA	0.31	USP1	0.29	TRAPPC8	0.28

TBC1D31	0.31	IPO7	0.29	DENND4C	0.28
LRRC8C	0.31	IDE	0.29	SMC3	0.28
USP9X	0.31	KIF5B	0.29	ATF1	0.28
EEA1	0.31	TXNDC9	0.29	NCBP1	0.28
NCKAP1	0.31	ATF7IP	0.29	SON	0.28
NEDD4	0.31	MYO9A	0.29	HSPH1	0.28
SLF1	0.30	U2SURP	0.29	ATP13A3	0.27
COPS2	0.30	ODR4	0.29	SLC25A46	0.27
PDCD10	0.30	GPD2	0.29	HECTD1	0.27
SUZ12	0.30	DICER1	0.29	SMARCA5	0.27
DPH3	0.30	SNX4	0.29	MPHOSPH9	0.27
HSP90AA1	0.30	BMPR1B	0.29	MYO5A	0.27
AKAP9	0.30	ATF6	0.29	MT-ND5	0.27
MTMR2	0.30	CPLANE1	0.29	PHTF2	0.27
BDP1	0.30	SCYL2	0.29	FEM1B	0.27
RPP30	0.30	NAA15	0.29	RBL1	0.27
PREPL	0.30	ACER3	0.29	GLS	0.27
DCTN4	0.30	ARL5B	0.29	REV1	0.27
MPHOSPH10	0.26	TMEM181	0.22	BCL3	-0.27
FBXO28	0.26	SRP72	0.22	CD151	-0.28
ITGAV	0.26	EIF1AX	0.20	IER5	-0.28
FAM208A	0.26	CKAP4	-0.21	CYHR1	-0.29
BPTF	0.26	EEF1A2	-0.22	ALDH3A1	-0.29
SLC26A2	0.26	APLP1	-0.22	REEP4	-0.29
FAM160B1	0.26	PKN1	-0.22	UNC119	-0.29
SENP6	0.26	TMED9	-0.23	NUDC	-0.29
ST6GAL2	0.26	HSPA2	-0.23	RTN4RL2	-0.29
SRSF1	0.26	LTBP3	-0.23	CYTH2	-0.29
IBTK	0.26	PNPLA2	-0.23	SCRN2	-0.29
SLC7A11	0.26	H2AFX	-0.23	BAIAP2	-0.29
ZRANB2	0.26	ZC3H4	-0.23	EHBP1L1	-0.29
INA	0.26	LLGL2	-0.24	SLC38A10	-0.29
ZFR	0.26	CTSL	-0.24	R3HDM4	-0.29
FGF2	0.26	LRPAP1	-0.24	AP5Z1	-0.30
LRPPRC	0.26	MGAT4B	-0.24	TOMM40	-0.30
YTHDF3	0.26	LTBP4	-0.24	SPHK2	-0.30
RAB21	0.26	LRP3	-0.25	AES	-0.30
GNG12	0.26	CYTH1	-0.25	ARFRP1	-0.30
BTBD7	0.26	MAF1	-0.25	SELENOO	-0.30
TTC37	0.26	FSTL3	-0.25	SSBP4	-0.30
PEG10	0.26	MAP3K12	-0.25	CFL1	-0.30
VPS13A	0.25	YIF1A	-0.25	NOMO2	-0.30
UBE2V2	0.25	MFGE8	-0.25	FJX1	-0.30
CSNK1G3	0.25	AXIN1	-0.26	GDF15	-0.31

PPP6R3	0.25	SRSF9	-0.26	IRF2BP1	-0.31
TRPM7	0.25	U2AF2	-0.26	B4GALNT4	-0.31
PAPOLA	0.25	TMEM8A	-0.26	RNPEPL1	-0.31
ZNF518A	0.25	DAPK3	-0.26	CEBPB	-0.31
SMC5	0.25	NOP53	-0.26	MRPL28	-0.31
TRAK2	0.25	KRT81	-0.26	KLF2	-0.31
SRGAP1	0.25	SIRT3	-0.26	HRAS	-0.31
DPY19L3	0.24	AKAP17A	-0.26	SPPL2B	-0.31
TLK1	0.24	CENPV	-0.27	PPP1R13L	-0.31
SOCS6	0.24	FRMD8	-0.27	PHLDA3	-0.31
ACSL3	0.24	BLVRB	-0.27	C1R	-0.31
ZNF24	0.24	NRSN2	-0.27	DEDD2	-0.31
CHORDC1	0.23	DDA1	-0.27	SNRNP70	-0.31
QKI	0.23	TSTA3	-0.27	FAAP100	-0.31
MRPL3	0.23	IGFBP4	-0.27	FP565260.1	-0.31
ZC3H15	0.23	SCYL1	-0.27	PKMYT1	-0.31
GMFB	0.23	LMAN2	-0.27	LSM4	-0.31
HERC4	0.22	FAM20C	-0.27	ORAI1	-0.31
ALDH16A1	-0.31	FUK	-0.35	CFD	-0.40
ECI1	-0.32	TNFRSF12A	-0.36	MAFF	-0.40
VWA1	-0.32	IGFBP6	-0.36	TRIM7	-0.40
SIRT7	-0.32	HDGFL2	-0.36	B3GNT9	-0.41
FIS1	-0.32	ANKRD9	-0.36	LMTK3	-0.41
WDR18	-0.32	ASPSCR1	-0.36	MPG	-0.41
ATP6V0B	-0.32	JUNB	-0.36	MNX1	-0.41
PRXL2B	-0.32	FTH1	-0.36	METRN	-0.41
AGPAT2	-0.32	SAMD11	-0.36	ARHGEF4	-0.41
ESRRA	-0.32	ZNF865	-0.36	ZNF385A	-0.41
NR2F6	-0.32	NSMCE3	-0.36	PABPN1	-0.41
CTSD	-0.32	TEDC2	-0.36	HAPLN3	-0.42
GIGYF1	-0.32	FAM214B	-0.36	CYBA	-0.42
YIF1B	-0.32	RRS1	-0.37	DDIT4	-0.42
PSRC1	-0.32	TMEM161A	-0.37	ATP5MF	-0.42
NFKBIA	-0.32	EML2	-0.37	CHST7	-0.42
ENGASE	-0.33	GNPTG	-0.37	MEX3D	-0.42
PRPF31	-0.33	IER5L	-0.37	MCRIP2	-0.42
ATP5F1D	-0.33	ACBD4	-0.37	MCRIP1	-0.42
HSPB1	-0.33	ARSA	-0.37	VEGFB	-0.43
TCEAL9	-0.33	SH3BGRL3	-0.37	CITED4	-0.43
SLC29A4	-0.33	EGFL7	-0.37	OLFM2	-0.43
ZNF316	-0.33	SNAPIN	-0.37	RBFOX3	-0.43
FBXL19	-0.33	CAPG	-0.37	NOMO3	-0.43
EPOP	-0.33	HOXB8	-0.38	ALKBH7	-0.43
SLC9A3R2	-0.33	JUND	-0.38	TRADD	-0.43

SIL1	-0.33	C19orf24	-0.38	MYPOP	-0.44
PRDX5	-0.34	PIDD1	-0.38	AURKAIP1	-0.44
ZNF707	-0.34	MFSD3	-0.38	PHLDA2	-0.44
OGFR	-0.34	CTXN1	-0.38	LFNG	-0.44
MAGEF1	-0.34	SARM1	-0.38	FAM131C	-0.44
RNF126	-0.34	ABHD17A	-0.38	PLXNA3	-0.44
SLC16A3	-0.34	PALM	-0.38	CORO6	-0.44
DECR2	-0.34	ADAMTS10	-0.38	H1FX	-0.45
SLC16A4	-0.34	TMEM187	-0.38	CCDC124	-0.45
PBXIP1	-0.35	TMEM250	-0.39	KRT86	-0.45
MAPK8IP3	-0.35	TSPAN9	-0.39	JOSD2	-0.45
PRR5	-0.35	GRB7	-0.39	JDP2	-0.46
TRIR	-0.35	SLC27A1	-0.39	FLYWCH2	-0.46
UBALD2	-0.35	MAZ	-0.39	ZBTB48	-0.46
USF1	-0.35	IER2	-0.40	COL16A1	-0.46
DUT	-0.35	ACOT2	-0.40	BAD	-0.47
ZNF444	-0.35	HCFC1R1	-0.40	ZNF703	-0.47
TMUB2	-0.35	SEMA3F	-0.40	TNNT1	-0.47
PPDPF	-0.47	CBWD3	-0.56	SMIM5	-0.95
DPP7	-0.47	TAF10	-0.56	SPDEF	-0.96
CACFD1	-0.47	ZBTB42	-0.57	MT-ATP8	-0.98
INCA1	-0.47	ENDOG	-0.57	GLUD2	-0.99
CTU1	-0.47	ADAM8	-0.57	RAB43	-1.02
ZFPM1	-0.48	ALDOA	-0.57	ATP2C2	-1.03
FAM171A2	-0.48	ARHGEF18	-0.57	TMC8	-1.05
IL11	-0.48	CHCHD10	-0.58	CRLF2	-1.20
MCAT	-0.48	GET4	-0.58	C10orf55	-1.26
GRIN3B	-0.48	BBC3	-0.58	SNAI3	-1.26
ZNF513	-0.48	OCEL1	-0.60	C1QTNF12	-1.32
PCSK1N	-0.49	PRRX2	-0.60	GOLGA8K	-1.33
CCDC24	-0.49	CBS	-0.60	B3GALT4	-1.42
SRXN1	-0.49	EXOC3L4	-0.61	TBC1D3G	-1.42
RPP25L	-0.49	CHRNA7	-0.62	PRRG2	-1.48
HOMER3	-0.49	AXIN2	-0.66	ZMAT4	-1.60
CBARP	-0.49	CLDN3	-0.67	DMRTA2	-1.76
NAT14	-0.49	PAX6	-0.69	PSG5	-2.14
SLC9A5	-0.51	TMEM238	-0.74	COL22A1	-2.31
CXCL3	-0.52	MDK	-0.78	DNAJC28	-2.41
DOHH	-0.52	ZNF57	-0.85	SERPINC1	-3.49
PHLDB3	-0.53	GPC2	-0.92	FBLL1	-4.17
IRX2	-0.53	MRPL23	-0.92	SLC1A2	-4.31
PAQR6	-0.53	SOX8	-0.93	LTA	-4.31
PDRG1	-0.55	BCL11B	-0.95	ADAMTS6	-4.56
SH3TC1	-0.55				

2. Differentially expressed genes after treatment by tyrosol ($p < 0.05$)

Gene name	Log2 Fold change	Gene name	Log2 Fold change	Gene name	Log2 Fold change
TACR2	4.13	BEST3	1.92	ZNF490	1.54
PRSS35	3.94	HSPA6	1.82	SLC25A21	1.47
C4BPB	3.44	RGPD5	1.75	NKAIN3	1.26
CBSL	3.17	IGSF9	1.62	MGAT2	1.12
ARL17B	1.07	NDUFA9	0.29	TPRA1	0.25
PPP1R16B	0.84	TIMM8A	0.29	SRSF9	0.25
PTAFR	0.83	SOD2	0.29	TNFAIP3	0.25
PTP4A1	0.76	4-Mar	0.28	METTL13	0.24
APLN	0.70	DUS3L	0.28	MAP4K5	0.24
SRXN1	0.63	OSTM1	0.28	SGMS1	0.24
PSD4	0.60	PGAM5	0.28	CENPN	0.24
CHRFAM7A	0.56	THOC3	0.28	RAB5C	0.24
TAF10	0.52	TIMM23	0.28	GPRIN2	0.24
CLMP	0.49	SH2D5	0.28	C18orf25	0.24
SP6	0.48	FRMD6	0.28	PREB	0.24
TXNDC12	0.47	RPS6KA4	0.28	ANAPC13	0.23
C19orf71	0.46	IGFBP7	0.28	SLC36A4	0.23
SESN2	0.45	MAPK14	0.28	RALA	0.23
GREB1L	0.45	AAMP	0.27	RPL8	0.23
RFTN1	0.45	EIF6	0.27	RNASEH1	0.23
TMEM258	0.43	SENP3	0.27	SAPCD2	0.23
KBTBD6	0.40	NAF1	0.27	NARS2	0.23
MRPL54	0.40	TATDN2	0.27	CCNC	0.23
CACTIN	0.39	CCSAP	0.27	ODC1	0.23
ROMO1	0.38	GSKIP	0.27	NIPA2	0.23
TGFA	0.37	C7orf50	0.27	DHCR24	0.23
HDAC11	0.37	SUV39H2	0.26	CD2BP2	0.23
B3GNT5	0.36	GTF2H3	0.26	OGFRL1	0.23
MARS2	0.36	SDHD	0.26	EIF4H	0.23
CCDC115	0.36	CARNMT1	0.26	SPCS3	0.23
RAB32	0.36	KRT10	0.26	DDHD1	0.23
MSTO1	0.36	PLEKHA8	0.26	NIP7	0.23
PRELID1	0.35	KIAA1549L	0.26	CA8	0.23
FAM207A	0.34	TFIP11	0.25	FXR2	0.23
ECHDC3	0.34	LRRC42	0.25	GALK1	0.22
POP7	0.33	BLOC1S6	0.25	CDC73	0.22
STK17B	0.32	EREG	0.25	PPP3R1	0.22
SELENOI	0.32	IMP4	0.25	TOR4A	0.22
RPP14	0.32	RRP9	0.25	SNX8	0.22

STUB1	0.31	SLC30A1	0.25	DYRK2	0.22
SLC5A3	0.31	TEX264	0.25	NCEH1	0.22
ENOX2	0.31	MYBL1	0.25	EIF4E2	0.22
RMDN3	0.31	CHTF8	0.25	ZNF526	0.22
SSX2IP	0.30	LAMC2	0.25	FEM1B	0.22
TRIM35	0.30	HNRNPLL	0.25	USP12	0.22
DCTPP1	0.30	APEH	0.25	CCDC85C	0.22
DNAJC14	0.29	ABHD13	0.25	URB2	0.22
EXTL2	0.29	OTUD7B	0.25	TWSG1	0.22

HSPA1B	0.21	ARL4C	0.19	CDC20	0.16
FGF2	0.21	TMX1	0.19	VKORC1L1	0.16
DYNLL2	0.21	SLC39A14	0.19	PCBP1	0.16
SPIRE1	0.21	PPIF	0.18	TARDBP	0.16
TMTC3	0.21	HMGCS1	0.18	EHD1	0.16
NAA50	0.21	C8orf33	0.18	EIF5	0.16
HNRNPD	0.21	EHBP1L1	0.18	STRAP	0.16
AXL	0.21	WDR6	0.18	LASP1	0.15
TMEM245	0.21	SHOC2	0.18	EIF3B	0.15
SLC35B2	0.21	NUP58	0.18	RBM3	0.15
WDR3	0.21	DDX1	0.18	PGK1	0.14
KLF16	0.20	EIF1AX	0.18	ATP5F1A	0.14
EIF5A	0.20	AGPAT5	0.18	ATP5F1B	0.13
PSMC1	0.20	SZRD1	0.18	HSPA8	0.13
SLC4A7	0.20	FN3KRP	0.18	ABCC2	-0.13
PCGF5	0.20	AHCY	0.18	SF3B4	-0.14
HSBP1	0.20	SNX17	0.18	ATXN2L	-0.14
ALDH1B1	0.20	ZC3H7B	0.18	UBC	-0.15
HNRNPAB	0.20	CDKN1A	0.18	CLU	-0.15
IRAK1	0.20	SINHCAF	0.18	GRAMD1A	-0.16
SLC16A1	0.20	POLR2C	0.18	LAMA3	-0.16
MAD2L1	0.20	UNG	0.18	BHLHE40	-0.17
RASSF3	0.20	RRM2	0.18	EPHX1	-0.17
ERI1	0.20	CCT4	0.17	DNAJB12	-0.17
NAA15	0.20	HPGD	0.17	DNM1	-0.17
SOCS6	0.20	SEMA3C	0.17	TSKU	-0.17
SORD	0.20	CNDP2	0.17	RTN4RL2	-0.18
NEDD4	0.20	GMFB	0.17	GCC2	-0.19
CYP1B1	0.19	ADAM9	0.17	PPDPF	-0.19
SETD7	0.19	PSMD2	0.17	PLEKHG2	-0.19
GHITM	0.19	UBE2M	0.17	BCKDK	-0.19
GTF2A1	0.19	CAVIN1	0.17	MYRF	-0.19
MTDH	0.19	CCDC47	0.17	PNISR	-0.20
GOT1	0.19	HSPH1	0.17	SIM2	-0.20

MYEOV	0.19	RPN1	0.17	FOXA2	-0.20
DHX36	0.19	HNRNPA0	0.17	GABRE	-0.20
MEPCE	0.19	SLC4A2	0.17	SREBF1	-0.20
CXCL5	0.19	FUBP3	0.17	MT-ATP6	-0.20
NMD3	0.19	ARF6	0.16	KANSL1	-0.20
IRS1	0.19	PTPN11	0.16	SYVN1	-0.20
SLC25A11	0.19	TSR1	0.16	ADGRG1	-0.21
PRMT5	0.19	BZW1	0.16	IER5L	-0.21
PFKP	0.19	NACC2	0.16	TIMP1	-0.22
DNAJC5	0.19	TMEM30A	0.16	NOMO2	-0.22
KCTD13	-0.22	OLFM2	-0.32	SPDEF	-0.60
TRAPPC1	-0.22	MT-ND5	-0.32	PAX6	-0.66
JUND	-0.22	MSANTD2	-0.33	MT-ATP8	-0.66
BCL3	-0.22	LRRC37B	-0.33	TBX19	-0.73
ATXN2	-0.22	MT-ND4	-0.34	TTLL6	-0.73
PEX26	-0.22	KIZ	-0.34	IRF9	-0.78
PAN2	-0.23	CORO6	-0.34	ETV2	-0.79
FTL	-0.23	RPS23	-0.35	NPIPB3	-0.79
SF3B6	-0.23	SYT17	-0.35	ALDOA	-0.79
SLC9A1	-0.24	OCEL1	-0.35	GLUD2	-0.80
NPIPB5	-0.24	HELB	-0.36	RORA	-0.87
CLCN6	-0.24	MT-ND2	-0.36	TMPRSS9	-0.89
TGFB1I1	-0.24	OSBPL7	-0.36	NKPD1	-0.95
CEACAM6	-0.24	MT-ND1	-0.38	TCAP	-0.95
CLSTN3	-0.24	EIF5AL1	-0.38	RAB40A	-0.97
CLK2	-0.25	HOMEZ	-0.38	NPIPB13	-1.00
ADAMTS10	-0.25	MT-CO1	-0.39	ENTPD8	-1.06
PROSER3	-0.25	SEC31B	-0.39	COLCA2	-1.10
MT-ND3	-0.25	SARM1	-0.39	GOLGA8K	-1.16
TMSB4X	-0.26	MT-CYB	-0.41	NUTM2G	-1.36
PTMS	-0.26	KLF9	-0.41	CDKN1C	-1.42
RAD9A	-0.26	MT-CO3	-0.42	DEPP1	-1.49
TCIM	-0.26	ANKRD36B	-0.42	MAN1C1	-1.53
DYRK1B	-0.26	INCA1	-0.43	LRRC19	-1.59
FBXL20	-0.26	UNC13A	-0.43	CARNS1	-1.61
PNRC1	-0.27	MERTK	-0.45	RGCC	-1.67
ATP2A3	-0.27	EXOC3L4	-0.45	EPHA6	-1.70
TMEM138	-0.27	UBE2V1	-0.45	SIRT4	-1.76
RABL2B	-0.27	SULT2B1	-0.47	WFIKKN1	-1.77
RTL8A	-0.27	ARL17	-0.47	ANKRD20A3	-1.84
FXYD5	-0.28	MT-CO2	-0.49	GOLGA8M	-2.09
DYNLRB1	-0.28	CBS	-0.50	GFI1B	-2.37
LTB4R	-0.28	ZRSR2	-0.50	PWP2	-2.37
MICAL1	-0.29	HOXB8	-0.50	METTL7A	-2.38

TTC9C	-0.29	CCDC78	-0.51	PRODH	-2.65
CCDC77	-0.29	COL1A1	-0.53	HINT2	-2.76
RBFOX3	-0.30	PLEKHO1	-0.55	CYP2A7	-3.53
PPP1R13L	-0.30	IGSF10	-0.56	PCDH17	-4.02
ZNF76	-0.30	MT-ND4L	-0.57	LIN28A	-4.07
MZF1	-0.31	FGA	-0.57	OTOGL	-4.07
PPARD	-0.32	OSR2	-0.58	PLEKHS1	-4.22
MTERF3	-0.31	IL3RA	-0.60	KRT23	-4.34
